# Nucleoside-Lipid-Based Nanoparticles for Phenazine Delivery: A New Therapeutic Strategy to Disrupt Hsp27-eIF4E Interaction in Castration Resistant Prostate Cancer

**DOI:** 10.3390/pharmaceutics13050623

**Published:** 2021-04-27

**Authors:** Hajer Ziouziou, Clément Paris, Sébastien Benizri, Thi Khanh Le, Claudia Andrieu, Dang Tan Nguyen, Ananda Appavoo, David Taïeb, Frédéric Brunel, Ridha Oueslati, Olivier Siri, Michel Camplo, Philippe Barthélémy, Palma Rocchi

**Affiliations:** 1 Centre de Recherche en Cancérologie de Marseille, CRCM, Inserm UMR 1068, CNRS, UMR 7258, Aix-Marseille University U105, Institut Paoli-Calmettes, F-13009 Marseille, France; zhajer24@yahoo.com (H.Z.); clement.paris@inserm.fr (C.P.); khanh.le-thi@inserm.fr (T.K.L.); andrieu.claudia@gmail.com (C.A.); nguyendangtancdy@gmail.com (D.T.N.); david.taieb@ap-hm.fr (D.T.); 2 Unit of Immunology Microbiology Environmental and Carcinogenesis (IMEC), Science Faculty of Bizerte, University of Carthage, 7000 Bizerte, Tunisia; ouelsatiridha12@hotmail.fr; 3 ARNA Laboratory, INSERM U1212/CNRS, UMR 5320, University of Bordeaux, F-33076 Bordeaux, France; sebastien@benizri.fr (S.B.); anandaappavoo@yahoo.fr (A.A.); 4 Biophysics and Nuclear Medicine Department, La Timone University Hospital, European Center for Research in Medical Imaging, Aix-Marseille University, F-13005 Marseille, France; 5 Centre Interdisciplinaire de Nanoscience de Marseille, Aix-Marseille University, CNRS, UMR 7325, 163, Avenue de Luminy, F-13288 Marseille, France; fred.brunel@gmail.com (F.B.); olivier.siri@univ-amu.fr (O.S.); michel.camplo@univ-amu.fr (M.C.)

**Keywords:** nucleolipid, dialkoxyphenazine, nanoformulation, prostate cancer

## Abstract

Heat shock protein 27 (Hsp27) has an established role in tumor progression and chemo-resistance of castration-resistant prostate cancer (CRPC). Hsp27 protects eukaryotic translation initiation factor 4E (eIF4E) from degradation, thereby maintaining survival during treatment. Phenazine derivative compound #14 was demonstrated to specifically disrupt Hsp27/eIF4E interaction and significantly delay castration-resistant tumor progression in prostate cancer xenografts. In the present work, various strategies of encapsulation of phenazine #14 with either DOTAU (N-[5′-(2′,3′-dioleoyl)uridine]-N′,N′,N′-trimethylammonium tosylate) and DOU-PEG_2000_ (5′-PEG2000-2′,3′-dioleoyluridine) nucleolipids (NLs) were developed in order to improve its solubilization, biological activity, and bioavailability. We observed that NLs-encapsulated phenazine #14-driven Hsp27-eIF4E interaction disruption increased cytotoxic effects on castration-resistant prostate cancer cell line and inhibited tumor growth in castration-resistant prostate cancer cell xenografted mice compared to phenazine #14 and NLs alone. Phenazine #14 NL encapsulation might represent an interesting nanostrategy for CRPC therapy.

## 1. Introduction

Prostate cancer (PC) is the fifth leading cause of death from cancer in men worldwide, with an estimated 307,000 deaths representing 6.6% of total male cancer mortality [1]. Most patients with advanced PC are treated with androgen deprivation therapy (ADT, also called castration). Despite the initial response to ADT, patients will experience disease progression to a castration resistant (CRPC) state. Patients with CRPC currently have few treatment options, and there is an unmet medical need in this area for new compounds that target the cancer differently and offer alternative therapeutic options for patients at this late stage of PC. Several therapeutic agents (abiraterone acetate, enzalutamide, docetaxel, cabazitaxel, radium-223, and sipuleucel-T) were found to improve overall survival and have been introduced in the management of metastatic CRPC. A combination of docetaxel and prednisone has been the first-line, standard-of-care cytotoxic therapy for metastatic castration-resistant prostate cancer (mCRPC) since 2004, following the demonstration of a survival benefit in 2 pivotal phase-3 trials [2,3] and, more recently, in combination with androgen deprivation therapy (ADT) without prednisone for castration-sensitive disease based on the chemohormonal therapy (ADT plus docetaxel) vs. ADT randomized trial for extensive disease in prostate cancer (CHAARTED) and systemic therapy in advancing or metastatic prostate cancer: Evaluation of Drug Efficacy (STAMPEDE) trial results [4,5]. Overall survival of mCRPC was found to be increased under abiraterone treatment compared to placebo (median 15.8 vs. 11.2 months, respectively, *p* < 0.0001) [6]. Very recently, therapeutic nuclear medicine using ^177^Lu-PSMA-617 has shown very promising effects in mCRPC progressing after docetaxel and could represent an alternative to cabazitaxel [7]. A docetaxel nanoparticle targeting PSMA also offers new opportunities for mCRPC with toxicity advantage over docetaxel-based chemotherapy [8].

**Figure 1:**
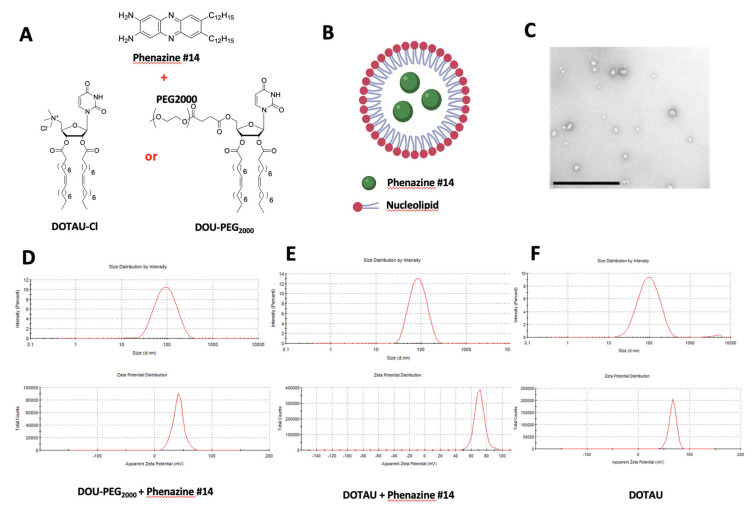
(**A**) Phenazine #14 formulation with DOU-PEG2000 (N-[5′-(2′,3′-dioleoyl)uridine]-N′,N′,N′-trimethylammonium tosylate)or DOTAU (5′-PEG2000-2′,3′-dioleoyluridine). (**B**) Schema of phenazine #14 (in green) encapsulation with the nucleolipids (not to scale). (**C**) TEM image showing NP_DOU-PEG2000_ (bar = 1 µM). (**D**) Dynamic light scattering experiment recorded on NPDOU-PEG2000 + phenazine 14 samples showing objects of 78.6 nm in diameter (polydispersity index of 0.222). Zeta potential of these objects, 41.2 mV (Zeta deviation 9.41). (**E**) Dynamic light scattering experiment recorded on NPDOTAU+ phenazine 14 samples showing objects of 74.9 nm in diameter (polydispersity index of 0.182). Zeta potential of these objects, 71.1 mV (Zeta deviation 6.37). (**F**) Dynamic light scattering experiment recorded on NPDOTAU samples showing objects of 83.8 nm in diameter (polydispersity index of 0.257). Zeta potential of these objects, 67.5 mV (Zeta deviation 6.88).

The small heat shock protein 27 (Hsp27), which is an ATP-independent molecular chaperon, has been implicated in PCa progression and resistance to hormone suppression and chemotherapy at latter stages (i.e., CRPC). We have previously shown that Hsp27 knockdown via an antisense oligonucleotide (called OGX-427) delayed progression in mice bearing LNCaP and PC-3 xenografts and restored the sensitivity of castration and chemotherapy [9,10]. A randomized phase-2 study of Hsp27 targeting antisense (apatorsen) with prednisone was associated with significant PSA (prostate-specific antigen) declines (≥50% in 47% of cases) compared to prednisone alone in metastatic CRPC [11]. Further evaluation of Hsp27 targeting with various formulations, doses, and combinations are warranted for improving clinical results. In order to improve understanding of Hsp27’s mechanisms of action, characterize additional putative cell survival proteins, and improve understanding of apoptotic regulatory pathways and drug resistance, we identified Hsp27 as a modulator of the eukaryotic translational initiation factor (eIF4E) and established a potential mechanism for the eIF4E-regulated apoptosis after androgen ablation and chemotherapy. We previously showed that Hsp27 drives treatment-resistance by protecting major client proteins such as eIF4E from their ubiquitin-proteasome degradation [12,13]. eIF4E binds the m7GTP cap structure at the 5′-end of mRNAs, stimulating the translation of proteins implicated in cancer cell growth and metastasis [14]. Hsp27-eIF4E confers resistance to therapies in PC cells via maintenance of protein synthesis and survival during androgen ablation and chemotherapy. eIF4E is a notoriously challenging target, and most of the reported inhibitors have limited effects [12]. Therefore, targeting Hsp27-eIF4E interaction may serve as a relevant therapeutic approach in advanced PC. In our previous work, we have shown that 2,3-Dialkoxyphenazines lipidic derivative inhibited Hsp27/eIF4E protein [15]. Screenings on 8 phenazine derivative compounds were based on bioluminescence resonance energy transfer (BRET) assays and validated by Co-Immunoprecipitation (Co-IP). The phenazine #14 derivative, a non-intercalating agent, has been identified as the most active candidate for inhibiting the Hsp27/eIF4E protein interaction and increasing cell apoptosis of PC-3 cells [15]. Although phenazine #14 showed inhibition of PC-3 cell proliferation, its solubility in biological fluids remains low (hydrophobic molecule; 1 mg/mL in aqueous buffer). Nanoparticle drug delivery systems are engineered technologies that use nanoformulation for the delivery and controlled release of therapeutic agents. We hypothesized that nanocarriers involving nucleolipids (NLs) could improve the solubility of phenazine #14 derivative and its intracellular delivery. As previously reported, hybrid nucleoside-lipid amphiphiles, often referred to as nucleolipids, have been successfully used as new pharmaceutical formulations, including nanoemulsions [16], lipid nanoparticles [17], or gels [18], for example. Due to their non-toxic nature and self-assembly properties, nucleolipids are ideally placed for encapsulating hydrophobic drugs with an expected improvement of drug bioavailability for cancer cells and subsequent increased antitumoral activity. In this contribution, two nucleolipids were chosen, DOTAU and DOU-PEG_2000_ (PEG tail is covalently grafted at the 5′ end of the nucleolipid) (Figure 1), for formulating the phenazine #14 in nanoparticles (NPs). DOTAU (N-[5′-(2′,3′-dioleoyl)uridine]-N′,N′,N′-trimethylammonium tosylate), a cationic nucleolipid, has been described to form supramolecular organization in aqueous solution. PEG (polyethylene glycol) formulation was tested since the presence of the PEG chain enhances particle solubility and stability, reduces renal clearance, increases half-life, lowers toxicity, and reduces immunogenicity [19]. Cationic nucleolipid properties lead to molecular interactions such as hydrogen bonding and π-π stacking that modulate the interactions between molecules and the synthetic vector. The structure formed with nucleolipids has shown important stability [20]. In addition, DOTAU has shown a good capacity to transfect GFP plasmid reporter in cell culture without inhibiting cell proliferation and exhibiting less toxicity than commercial transfection reagents [21]. The other nucleoside-based lipid, 5′-PEG2000-2′,3′-dioleoyluridine (DOU-PEG_2000_), has been evaluated as micellar nanoparticles for cell transfection. In this study, the internalization of the resulting micelles was facilitated in the presence of serum in ovarian cancer cells (SKOV3) [22].

## 2. Materials and Methods

### 2.1. Cell Lines and Cell Culture

The androgen-independent prostate cancer cell line PC-3 was purchased from the American Type Culture Collection (ATCC, Manassas, VA, USA) and maintained in Dulbecco’s Modified Eagle’s Medium (Life Technology, Inc., Saint Aubin, France) supplemented with 10% fetal bovine serum (FBS). PC-3 were cultivated at 37 °C in 5% CO_2_.

### 2.2. Phenazine Derivative Compound#14

The phenazine derived compound #14 (phenazine #14) was provided by Drs. M. Camplo and O. Siri (Marseille Interdisciplinary Center for Nanosciences, CNRS, UPR3118, Marseille, France). Phenazine #14 was dissolved in DMSO (dimethyl sulfoxide) at a stock concentration of 10 mM.

### 2.3. Nanoparticles DOTAU (NP_DOTAU_) Loading with Phenazine #14

Eight milligrams of DOTAU-Cl (Cas number: 868226-06-6; Mw: 790.2 Da) was solubilized in chloroform (200 µL) (DOTAU-Cl is fully soluble in dichloromethane) [21]. In another hemolyse tube, 8 mg of phenazine #14 was solubilized in chloroform (200 µL). After stirring, the nucleolipid solution was added to the drug solution. The solvent was removed to yield a nucleolipid film. The nucleolipid film was thoroughly dried to remove the residual organic solvent under vacuum for 2 h. The nucleolipid layer was then rehydrated in 4 mL of EuroBio Water (2 mg/mL). After sonication: 4 × 15 min (37 kHz, 100%, 26 °C), the particle size and zeta potential were determined by DLS (NanoZS, Malvern Zetasizer, Palaiseau, France) (20 μL in 400 μL Eurobio Water).

### 2.4. Nanoparticles DOU-PEG_2000_ (NP_DOU-PEG_) Loading with Phenazine #14

Eight milligrams of DOU-PEG_2000_ was solubilized in chloroform (200 µL). In another vial, 8 mg of compound#14 was solubilized in chloroform (200 µL). After stirring, the nucleolipid solution was added to the drug solution. After stirring, the mixed solution was then added dropwise in a large volume of water (15 mL of Eurobio Water). The resulting mixture was then concentrated and the remaining organic solvent was removed under reduced pressure. The final volume was then fixed at 4 mL (2 mg/mL). After sonication: 4 × 15 min (37 kHz, 100%, 26 °C), the particle size and zeta potential were determined by DLS (NanoZS, Malvern Zetasizer, Palaiseau, France) (20 μL in 400 μL Eurobio Water).

### 2.5. Transmission Electron Microscopy (TEM)

Nanoparticles were visualized by negative staining microscopy. Ten microliters of nanoparticles were transferred to a carbon-coated copper grid for 10 min. The sample was then dried and stained with 2.5% (*w*/*w*) of uranyl acetate in water for 2 min. The specimens were observed with a Hitachi H 7650 electron microscope.

### 2.6. Treatment of PC-3 Cells with the Phenazine #14 and the NPs

PC-3 cells were seeded into 10 cm dishes (1,250,000 cells/well) or 12-well plates (50,000 to 100,000 cells/well) according to the different experiments. The day after, the medium with 10% fetal bovine serum (FBS) was changed to a fresh one containing DMSO (control) or derivative of phenazine #14 or nucleolipids NP_DOTAU_ and NP_DOU-PEG_ at a concentration of 100 µM according to the different experiments. Effects of the treatment were analyzed 48 h later.

### 2.7. In Vivo Tumor Growth Evaluation

For in vivo study, 10^6^ PC-3 cells were inoculated in the flask region of 2-week- old male athymic mice (NOD SCID). (project update: #2020062314545490; 23 June 2020; ministère de la Recherche). Dr Palma Rocchi possesses personal agreement (#A13-477) for the animal handling and experimentation for this study. Mice were maintained in the animal facility (agreement #13.2700).

Tumor volumes were measured weekly with a caliper as follows: length × width × depth × 0.5236. When PC-3 tumors reached 300–500 mm^3^, mice were randomly selected for treatment with PBS (control) alone, phenazine #14 in PBS with 0.1% DMSO, DOTAU alone, and NP_DOTAU-phenazine #14_. Each experimental group consisted of 6 mice of control and 8 mice for other groups. Phenazine derivative phenazine #14 was tested at 1 mg/kg, which corresponded to its maximum of solubility, or NP_DOTAU-phenazine #14_ was tested at 2 mg/kg. The administration was done over 8 weeks with two injections per week. Data points were expressed as mean ± SEM.

### 2.8. Immunoprecipitation (IP)

The IP experiments were conducted as previously described [13]. Briefly, the protein quantification was done using Pierce BCA Protein assay (Thermo Fisher Scientific, Illkirch, France). The lysate (1 mg) was diluted in the lysis buffer to obtain the final volume of 400 µL and incubated with 8 µL (1/50) of rabbit anti-eIF4E antibody (Cell Signaling, Ozyme, Saint-Quentin-en-Yvelines, France) O/N at 4 °C. The immune complexes were precipitated by incubating with 30 µL of Trueblot anti-rabbit Ig IP beads (eBiosciences, Paris, France) for 1 h at 4 °C, and subsequently underwent 3-time wash with the cold lysis buffer. Eventually, the beads were re-suspended in 6 µL of protein sample buffer (Bio-Rad, Marnes-la-Coquette, France) and heated at 95 °C for 5 min prior to Western Blot analysis.

### 2.9. Western Blot

Western blot analyses were performed using a previously published method [15]. The following antibodies were used: rabbit Hsp27 antibody (Assay Designs, Villeurbanne, France, 1/5000), rabbit anti-eIF4E antibody (Cell Signaling, Ozyme, 1/1000, Saint-Cyr-l’école, France), anti-rabbit IgG HRP conjugate antibody (Santa Cruz Biotechnology, Heidelberg, Germany, 1/5000), and anti-rabbit Trueblot IgG HRP conjugate antibody (eBiosciences, 1/1000, Villebon-sur-Yvette, France). Loading levels were normalized using mouse anti-vinculin antibody (Sigma-Aldrich, 1/2000, Saint-Quentin-Fallavier, France). Re-blot Plus Mild Solution (Millipore, Molsheim, France) was used for membrane stripping during 9 min at RT.

### 2.10. Confocal Microscopy

PC-3 cells were seeded into a 12-well plate containing cover glasses covered by FBS, at a density of 100,000 cells/well. Twenty-four hours later, cells were treated with compounds (DMSO, DOU-PEG_2000_, DOTAU, phenazine #14, NP_DOU-PEG2000-phenazine #14,_ and NP_DOTAU-phenazine #14_) at 100 µM as indicated above. After 48 h of treatment, cells were washed with PBS1X and fixed with formaldehyde 4% (Thermo Fisher Scientific, Illkrich, France) for 15′ at RT. Subsequently, the cover glasses were washed with PBS 1 × 2 times and mounted on the glass slides using Prolong Gold antifade reagent with DAPI (Life Technologies, Villebon-sur-Yvette, France). The samples were kept to dry in the dark for 24 h, RT, and sealed with nail polish. Images were captured with Zeiss 510 META fluorescence confocal microscope plan 40×/1.4 (Carl Zeiss, Le Pecq, France) for both phenazine #14 (absorption; 452 nm, emission; 478 nm) and DAPI (absorption; 350 nm, emission; 450 nm–490 nm).

### 2.11. Cell Viability Assay

Cells were seeded in 12-well plates at the density of 50,000 to 100,000 cells/well. After 24 h, cells were treated with the derivatives of phenazine #14, NP_DOTAU-phenazine #14_, NP_DOU-PEG2000-phenazine #14_, DOTAU, and DOU-PEG_2000_ in a total volume of 500 μL/ well. After 48 h, cells were stained with 100 µL of MTT (3-(4,5-dimethylthiazol-2-yl)-2,5-diphenyl tetrazolium bromide) for 2 h at 37 °C in an atmosphere of 5% CO_2_. After drying, the cells are re-suspended in 500 µL of DMSO for 30 min at room temperature. The optical density of each well was then read at 595 nm by spectrometry. Each assay was performed in triplicate.

### 2.12. Flow Cytometry

PC-3 cells were seeded into 6 cm dishes at a density of 250,000 cells/well. After 24 h, cells were treated with phenazine #14, DOU-PEG_2000_, DOTAU, NP_DOU-PEG2000-phenazine #14,_ and NP_DOTAU-phenazine #14_ at 100 µM as indicated above. After 48 h of treatment, dead and living cells were collected and washed with PBS1X, before being fixed in cold ethanol 70% (250 µL/pellet). After 1 h incubation at 4 °C, cells were washed with PBS1X before permeabilization using flow buffer (8 parts of citric acid 0.1M, 192 parts of Na_2_HPO_4_ 0.2M) (100 µL/pellet). After another washing step, RNA was degraded by incubation (30 min at 37 °C) into PBS1X containing RNA-ase (Sigma-Aldrich, Saint-Quentin-Fallavier, France) at 0.5 mg/mL (200 µL/pellet). Supernatant was removed and pellet was incubated (30 min, RT, dark) in PBS1X containing propidium iodide at 0.05 mg/mL (1 mL/pellet) in order to stain DNA. Samples were transferred into FACS tubes and DNA content was determined by flow cytometry using LSRII SORP (Becton Dickinson, Le Pont de Claix, France) machine. Rates of apoptosis were then measured using FlowJo software (Tree Star, Inc, Ashland, United States).

### 2.13. Immunohistochemistry

The sections of paraffin-embedded tumors at 3 μm thickness were transferred onto the glass slides and allowed to dry overnight at RT. Prior to antibody staining, antigen retrieval to unmask the antigenic epitope was performed. For that, the slides were incubated at 65 °C (90 min), then at 95 °C (20 min) with EnVision FLEX Target Retrieval Solution (K8005; Agilent, Montpellier, France), followed by pretreating with Epitope Retrieval Solution (containing detergent; K5207; Dako UK Ltd., Ely, UK) for 30 min at RT. Subsequently, the samples were incubated with Dako EnVision FLEX Peroxidase- Blocking Reagent SM801 (ready to use) (K8000, K8002, K8023; Dako UK Ltd., Ely, UK) for 5 min in order to block endogenous peroxidase activity, which was followed by washing thoroughly using wash buffer FLEX (Dako UK Ltd.). The sections were incubated with the antibody, FLEX Monoclonal Mouse Anti-Human Ki-67 (clone MIB-1, IR62; Dako UK Ltd., Ely, UK), for 1 h at RT, followed by washing 3 times with FLEX buffer. The slides were incubated with Dako EnVision FLEX/HRP SM802 (K8000; Dako UK Ltd., Ely, UK) for 25 min at RT and then washed 3 times with FLEX buffer. To reveal the color of antibody staining, diaminobenzidine substrate solution (Dako UK Ltd., Ely, UK) was applied to the sections on the slides and incubated for 10 min at RT. The slides were washed with FLEX buffer, counterstained with EnVision FLEX HEMATOXILIN SM806 (K8008; Dako UK Ltd., Ely, UK) for 5 min, followed by one wash with FLEX buffer and with water. Finally, the samples were dehydrated, cleared, and mounted with aqueous mounting media (LEICA AUTOSTAINER JUNG XL, Leica, Nanterre, France). Positive and negative controls were carried out with each batch of slides.

### 2.14. Statistical Analysis

Statistical analyses were conducted using one-way analysis of variance followed by Fisher’s protected least significant difference test (Statview 512, Brain Power Inc., Calabases, CA, USA). Mean +/− SEM represents results. *: *p* ≤ 0.05, **: 0.01, ***: *p* ≤ 0.001.

## 3. Results

The aim of the study was to develop DOTAU and DOU-PEG_2000_ based nanocarriers facilitating phenazine delivery in order to improve its solubility, cell penetration, and therapeutic effect in CRPC.

In order to obtain stable nanoparticles loaded with phenazine derivative, we developed a simple encapsulation procedure by dissolving a nucleolipid/phenazine #14 film in an aqueous medium. As expected, the weak interactions (hydrophobic effect, hydrogen bonding, π-π stacking) occurred between the nucleobases of the nucleolipids and the phenazine #14 [17]. Nanoparticles composed of each NP (DOTAU and DOU-PEG_2000_ and phenazine #14 were established and referred to as NP_DOTAU-phenazine #14_ and NP_DOU-PEG2000-phenazine #14,_ respectively (Figure 1). In accordance with dynamic light scattering (DLS) data, transmission electron microscopy (TEM) images showed oblong monodisperse objects of 74.9 nm (polydispersity index 0.182) diameter for NP_DOTAU-phenazine #14_ and 78.6 nm (polydispersity index 0.222) diameter for NP_DOU-PEG2000-phenazine #14_ (see example of NP_DOU-PEG2000_ in Figure 1). Zeta-potential measurements showed positive values of 71.1 mV (Zeta deviation 6.37 mV) for NP_DOTAU-phenazine #14_ and 41 mV (Zeta deviation 9.41 mV) for NP_DOU-PEG2000-phenazine #14_ nanoparticles, indicating that positive or neutral nucleolipids wrapped the NP. The encapsulation of phenazine in DOTAU (Figure 1E) did not induce significant changes regarding the size distribution or the zeta potential compared with DOTAU alone (Figure 1F).

**Figure 2:**
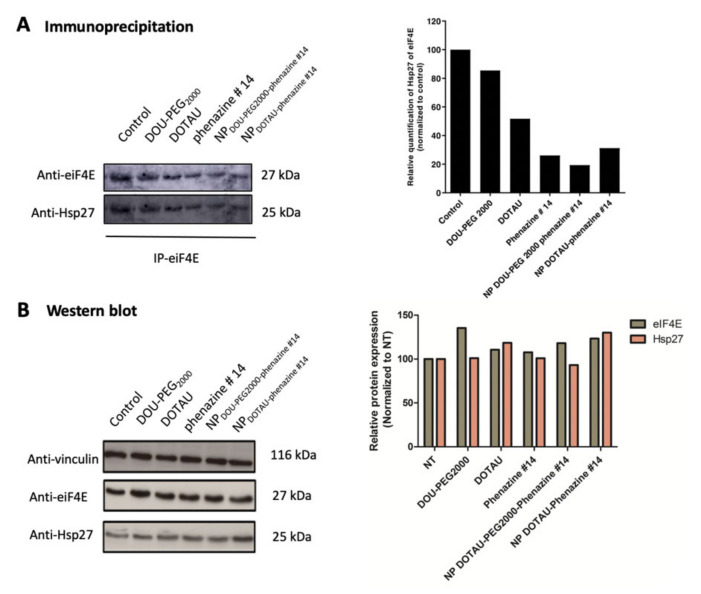
The encapsulated phenazine #14 with DOTAU and DOU-PEG_2000_ at 100 µM inhibits the interaction of eIF4E/Hsp27. (**A**) Co-immunoprecipitation of eIF4E showing the interaction of eIF4E and Hsp27, (**B**) Western Blot analysis showing the expression levels of eIF4E and Hsp27 compared to vinculin.

PC-3 cells were treated with phenazine #14, DOTAU, DOU-PEG_2000_, NP_DOTAU-phenazine #14_, and NP_DOU-PEG2000-phenazine #14_ at 100 μM. Co-immunoprecipitation analyses showed that NP_DOTAU-phenazine #14_ and NP_DOU-PEG2000-phenazine #14_ inhibited eIF4E/Hsp27 interaction (Figure 2A), demonstrating that encapsulation maintains the drug activity. This effect was unrelated to protein degradation (Figure 2B).

**Figure 3:**
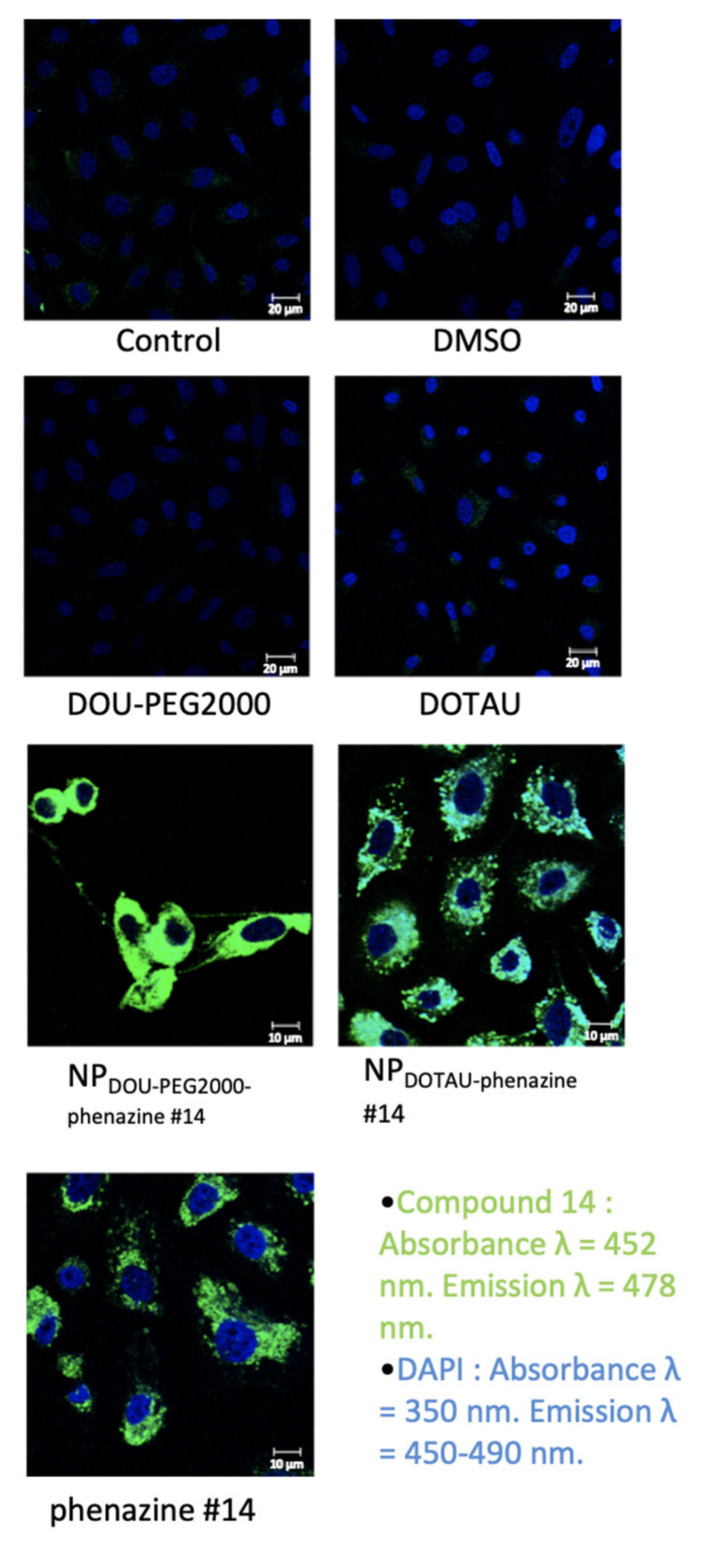
Confocal microscopic distribution of phenazine #14, NP_DOU-PEG2000-phenazine #14,_ and NP_DOTAU-phenazine #14_. PC-3 cells were treated at 100 µM with phenazine #14 (last panel), NP_DOTAU-phenazine #14_ (right panel), and NP_DOU-PEG2000-phenazine #14_ (left panel) with DMSO (upper right panel) as control. Auto-fluorescence of phenazine #14, NP_DOTAU-phenazine #14_, and NP_DOU-PEG2000-phenazine #14_ (green) and staining of the nucleus by DAPI (4′,6-diamidino-2-phenylindole, blue) was observed. PC-3 cells were treated with DMSO (control) during 48 h and proteins were extracted.

Using the auto-fluorescence property of phenazine #14, which absorbs at λ = 452 nm and emits a fluorescence at λ = 478 nm, we analyzed the intracellular distribution of DOU-PEG_2000_ and DOTAU by confocal microscopy. DOU-PEG_2000_ and DOTAU do not emit any fluorescence. Phenazine #14 was shown to be internalized in cells and mainly localized in the cytoplasm with a heterogeneous distribution. A previous study performed in our laboratory [15] identified phenazine #14 as an effective agent for Hsp27/eIF4E disruption that occurs in the cytoplasm. Confocal microscopy demonstrates that DOTAU-Cl and DOU-PEG encapsulation of the phenazine #14 does not modify the auto-fluorescence property, internalization, or cellular distribution of phenazine #14 (Figure 3). Phenazine 14 is not soluble in water or in 20/80 water/ethanol, whereas DOTAU and DOU-PEG formulations are fully soluble in water (Figure S1). The encapsulation of the phenazine #14 by DOTAU was only slightly modified compared to phenazine #14, with a pattern of aggregates. The phenazine #14 DOU-PEG_2000_ staining pattern was much more intense and homogenous that the latter ones (Figure 3).

**Figure 4:**
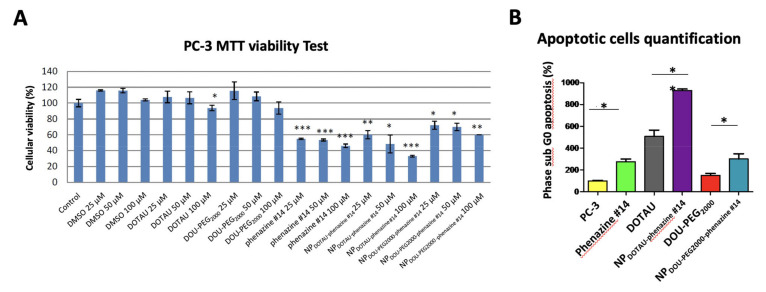
NP_DOTAU-phenazine #14_ inhibits cell viability and increases apoptosis of PC-3 cells in vitro. (**A**) MTT (3-(4,5-Dimethylthiazol-2-yl)-2,5-Diphenyltetrazolium Bromide) quantification of PC-3 cell viability was performed on cells treated with NP_DOTAU-phenazine #14_ and NP_DOU-PEG2000-phenazine #14_ at different concentrations (25, 50, 100), and NT (control) during 48 h. *, *p* ≤ 0.1, **, *p* ≤ 0.01 and ***, *p* ≤ 0.001. (**B**) Apoptotic cell quantification (SubG0 phase) by flow cytometry was performed on PC-3 cells treated with the compound NP_DOTAU-phenazine #14_ and NP_DOU-PEG2000-phenazine #14_ and NT (Non-Treated cells, control) at 100 µM during 48 h and labeled with propidium iodide. The bar graph represents cell percentage in subG0 phase. *, *p* ≤ 0.1, **, *p* ≤ 0.01 and ***, *p* ≤ 0.001.

NP cytotoxicity was assessed by MTT test and assessment of cellular morphology. DOTAU and DOU-PEG_2000_ at 100 µM concentration led to a 10% inhibition of cell proliferation compared to the control group. Higher cell apoptosis activity was observed with NP_DOTAU-phenazine #14_ compared to NP_DOU-PEG2000 -phenazine #14_ and in a dose-dependent manner. NP_DOTAU-phenazine #14_ inhibited proliferation of PC-3 by 65% at 100 µM, compared to 45% for 100 µM of phenazine #14 (Figure 4A). By contrast, NP_DOU-PEG2000 -phenazine #14_ was less effective. The effect of NP_DOTAU-phenazine #14_ was mainly driven by apoptosis, as shown by the high percentage of cells at subG0 phase of the cellular cycle (Figure 4B).

**Figure 5:**
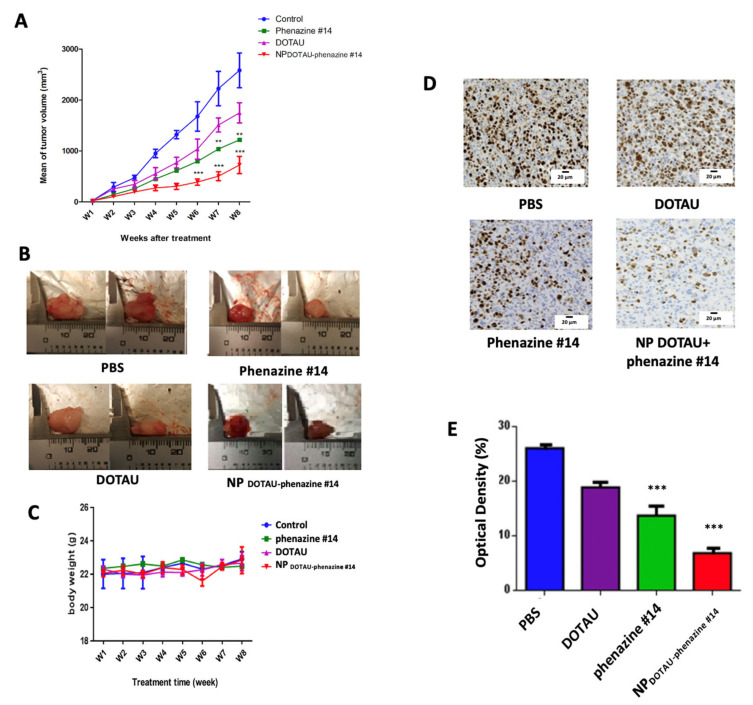
NP_DOTAU-phenazine #14_ decreased tumor volume in vivo. (**A**) NP_DOTAU-phenazine #14_ significantly enhanced anticancer activity in tumor-xenograft mice. NOD SCID mice were treated with free phenazine #14 (1 mg/kg), DOTAU, and NP_DOTAU-phenazine #14_ (2 mg/kg) via i.p. administration (twice per week, *n* = 8). PBS group was used as control (*n* = 6). Tumor volume was measured twice per week. Data are shown as mean ± SEM. (**B**) Representative pictures of the PC-3-derived xenograft tumors harvested from mice that received i.p. compound NP_DOTAU-phenazine #14_ or control-PBS, phenazine #14, and DOTAU after an 8-week treatment. (**C**) The body weight of the mice during the treatment time. Mouse body weight was measured twice per week. The graph shows that the treatments did not have side effects on body weight of the mice. Data are shown as mean ± SEM. (**D**) Ki-67 IHC staining of tumor tissues to assess tumor proliferation. (**E**) Distribution of tissue Ki-67 immunostaining intensity (measured as average optical density) according to the tumor treated with PBS, DOTAU, phenazine #14, and NPDOTAU-phenazine #14. Data are shown as mean ± SEM.

The in vivo test was carried out on mice xenografted with androgen-independent PC-3 prostate cancer cells. DOTAU, phenazine #14, and NP_DOTAU-phenazine #14_ were administered by i.p. twice a week for 8 weeks. At the end of the experiment after 8 weeks, we observed that 1- DOTAU cargo induced a 30% decrease of tumor growth, 2-phenazine #14 induced a 50% decrease of tumor growth, and 3- formulated NP_DOTAU-phenazine #14_ induced a 70% decrease of tumor growth. These results were consistent with in vitro experiments. The formulation of the NP_DOTAU-phenazine #14_ significantly reduced androgen-independent PC-3 tumor growth (*n* = 8, *** *p* ≤ 0.01) (Figure 5A,B; Tables S1–S4). The treatment was associated with a very good condition of mice compared to the control arm with no weight loss (Figure 5C). Therefore, no major toxicity is expected but further toxicity experiments would need to be performed prior to any clinical application. The assessment of Ki-67 immunoexpression and optical density in the harvested tumors was correlated with the inhibition of tumor growth (13.7 ± 0.18 for phenazine #14 vs. 6.81 ± 0.14 for NP_DOTAU-phenazine #14_, *p* < 0.0001) (Figure 5D,E).

Castration-resistant prostate cancer (CRPC) has always presented significant challenges to clinicians. In the last few years, several new options have been implemented in the therapeutic armamentarium against CRPC and clinical trials are underway to test the safety and effectiveness of novel androgen receptor (AR) targeting agents. The prognosis of CRPC remains poor and new therapeutic strategies are warranted. One of the main challenges is to find drugs with a favorable risk-benefit ratio. To overcome potential side effects, various nanovehicles such as polymeric micelles and vesicles [23], liposomes [24], and nanogels [25] have been developed. In parallel, bioinspired hybrid amphiphiles featuring both nucleosides as a lipid headgroup and lipophilic alkyl chains have recently emerged as promising molecules due to self-assembling nucleic acid-nucleolipid supramolecular systems [26] for improving drug [27] or nucleic acid delivery [28]. The molecular structure of the nucleolipids, including the base [29], the stereochemistry [30], and the charges [31], influences their drug delivery properties.

In our previous work, we have shown that phenazine derivative phenazine #14 specifically disrupted the Hsp27/eIF4E interaction, increased apoptosis, and decreased tumor growth [15]. The family of heterocyclic nitrogenous phenazine molecules have been originally extracted [32] and used as antibiotic [33], antimalarial [34], and anticancer agents [35] with limited side effects on healthy tissues [36]. 2,3-Dialkoxyphenazine lipidic derivatives have shown anticancer activities on pancreatic cancer cell lines [37]. Phenazine derivatives were also shown to induce apoptosis in human PC cells. Despite good biological activity, the main limitation of phenazine# 14 remains its poor solubility in aqueous buffer. In order to overcome the solubility issue, phenazine #14 was formulated with 2 cationic nucleolipids for encapsulation as a nanoparticle formulation. DOTAU [21] and DOU-PEG_2000_ [22] are a nucleoside-based cationic lipid derived from uridine.

The encapsulation of phenazine #14 with both NLs was carried out following the film hydration method, leading to obtaining monodisperse NPs without observing any aggregation during the process. The π-π stacking and hydrogen bonding interaction enhanced the stability of the formed NPs. In both cases, the second layer formed by the NL of the NP enhanced the water solubility either due to the positive charge of the DOTAU or the miscibility in water properties of the DOU-PEG_2000_. The analysis by dynamic light scattering showed the size of NPs to be around 75–78 nm, and their positive zeta potentials (+71 mV for NP_DOTAU-phenazine #14_ and + 42 mV for NP_DOU-PEG2000-phenazine #14_) indicated that the NLs surround the NPs.

We first confirmed that the nanoformulation did not impair the inhibition effect of phenazine #14 on Hsp27/eIF4E interaction. The proliferation studies demonstrated that NP_DOTAU-phenazine #14_ was the most effective compound in cell proliferation inhibition. The increased cellular uptake of NPDOTAU-phenazine #14 compared to NP_DOU-PEG2000-phenazine #14_ could be due to the positive charges at their surface (as shown in the zeta potential measurement) that may enhance interaction with cells and the cellular internalization process. NP_DOTAU-phenazine #14_ also demonstrated better pro-apoptotic activity compared to the other agents. The in vivo experiments were also in favor of NP_DOTAU-phenazine #14_ and showed impressive anti-tumor effects on PC-3 xenografts, which represents an aggressive androgen-independent prostate cancer model. Ki-67 was clearly decreased in the NP_DOTAU-phenazine #14_ arm, explained together with the increased pro-apoptotic effect of the therapeutic benefit. The positive effect is probably due to an increase of accumulation in the tumor area by passive targeting due to enhanced permeability retention (EPR) effect [38,39,40]. This behavior induces a local increase of drug concentration into the tumor, resulting in better antitumoral activity compared with free drugs. Furthermore, small-sized NPs are exceptionally favorable for anticancer drug distribution due to their ability to escape kidney excretion and spleen sequestration [41]. The NP_DOTAU-phenazine #14_ diameter (75 nm) probably influences tumoral size growth inhibition because it avoids phenazine #14 renal clearance.

Our results are in agreement with the study of Khiati et al. [42] which describes in vivo delivery profile of cisplatin formulated with NL. They showed an improved effect of nanoformulated cisplatin. They explained these results by the combination of 3 effects: decrease of cisplatin interaction with serum proteins, decrease of cisplatin renal clearance, and accumulation in liver and spleen. The expected advantage is to reduce toxicity, allowing increased doses in selected cases. Another study showed that an original nanomicellar drug delivery system based on an amphiphilic dendrimer (AmDM) could generate supramolecular micelles to effectively encapsulate the anticancer drug doxorubicin (DOX) with high drug-loading capacity. In this study, the NL increased the anti-tumor effect in doxorubicin-resistant MCF-7R cells [43] due to higher cellular uptake compared to free DOX and inhibited tumor growth at lower doses than free DOX.

## 4. Conclusions

Taken together, our results demonstrated that lipids featuring nucleoside moieties afford an efficient approach to address the delivery issues of hydrophobic drugs such as phenazine, including its low aqueous solubility and poor intracellular delivery. Furthermore, the present work demonstrated that NP_DOTAU-phenazine #14_ can disrupt Hsp27/eIF4E interaction and treat CRPC with a good clinical tolerance of preclinical models. Hence, nucleolipid-based nanoparticles appear as ideal candidates for use as a vehicle for phenazine delivery, as witnessed by the increased antitumor activities observed in vivo on PC-3 cell lines and in vivo on tumor-xenograft mice.

## Data Availability

The data presented in this study are available on request from the corresponding author.

## Supplementary Materials

The following are available online at https://www.mdpi.com/article/10.3390/pharmaceutics13050623/s1, Figure S1. Phenazine #14 Solubility. Phenazine 14 is not soluble in water and in 20/80 water/ethanol whereas DOTAU and DOU-PEG formulations are fully soluble in water. Table S1. Mice volume tumor size evolution (in mm^3^) during 8 weeks of treatment for the non-treated group. Table S2. Mice volume tumor size evolution (in mm^3^) during 8 weeks of treatment for the phenazine #14 group. Table S3. Mice volume tumor size evolution (in mm^3^) during 8 weeks of treatment for the DOTAU-Cl alone group. Table S4. Mice volume tumor size evolution (in mm^3^) during 8 weeks of treatment for the NP_DOTAU-phenazine #14_ group.

## Abbreviations

CRPC: castration-resistant prostate cancer; PC: prostate cancer; eIF4E: eukaryotic translation initiation factor 4E; NL: nucleolipids; ADT: androgen deprivation therapy; PSA: prostate-specific antigen.
