# Supplementary material for: Nucleoside-Lipid-Based Nanoparticles for Phenazine Delivery: A New Therapeutic Strategy to Disrupt Hsp27-eIF4E Interaction in Castration Resistant Prostate Cancer
Source: Pharmaceutics. 2021 Apr 27;13(5):623. doi: 10.3390/pharmaceutics13050623 (PMC8146835; doi:10.3390/pharmaceutics13050623)
Supplement: Supplementary file 1 [file pharmaceutics-13-00623-s001.zip › pharmaceutics-1126119-supplementary.pdf]

pharmaceutics

MDPI

Supplementary Materials: Nucleoside-Lipid-Based Nanoparticles for Phenazine Delivery: A New Therapeutic Strategy to Disrupt Hsp27-eIF4E Interaction in Castration Resistant Prostate Cancer

Hajer Ziouziou, Clément Paris, Sébastien Benizri, Thi Khanh Le, Claudia Andrieu, Dang Tan Nguyen, Ananda Appavoo, David Taïeb, Frédéric Brune, Ridha Oueslati, Olivier Siri, Michel Camplo, Philippe Barthélémy and Palma Rocchi

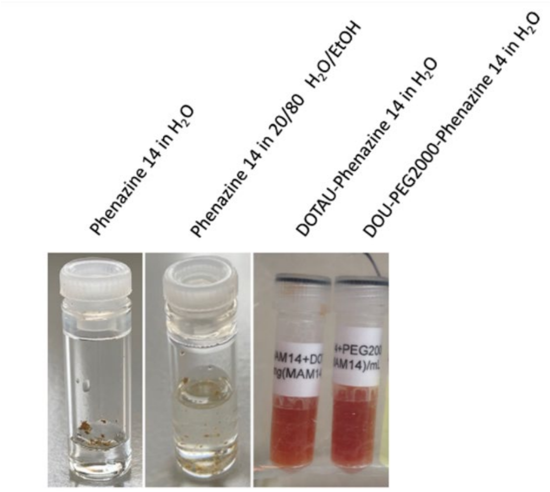

Figure S1. Phenazine # 14 Solubility. Phenazine 14 is not soluble in water and in 20/80 water/ethanol whereas DOTAU and DOU-PEG formulations are fully soluble in water.

Table S1. Mice volume tumor size evolution (in mm^3) during 8 weeks of treatment for the non-treated group.

Control									
**Week**	**Mouse Tumor Volume in mm3**						**Mean**	**SD**	**SEM**
	**1**	**2**	**3**	**4**	**5**	**6**			
1	65,5	44,7	31,3	21,6	0,0	8,0	28,5	24,2	9,9
2	188,8	125,4	717,3	268,1	317,9	132,0	291,6	221,9	90,6
3	625,1	345,8	616,9	367,1	418,6	474,7	474,7	121,8	49,7
4	1194,2	609,4	1098,8	844,8	1016,0	952,6	952,6	206,5	84,3
5	1212,5	1571,5	1553,5	1108,2	1167,2	1322,6	1322,6	198,7	81,1
6	1090,9	1746,1	1980,7	882,8	2853,4	1510,8	1677,4	704,7	287,7
7	1279,8	3379,6	1425,6	2954,1	2259,8	2058,3	2226,2	827,8	337,9
8	1494,1	2465,0	4071,0	2676,7	2338,3	2454,0	2583,2	836,6	341,5

Table S2. Mice volume tumor size evolution (in mm^3) during 8 weeks of treatment for the phenazine # 14 group.

Phenazine #14		
**Week**	**Mouse Tumor Volume in mm3**	**Mean**

	**1**	**2**	**3**	**4**	**5**	**6**	**7**	**8**	
1	36,6	17,9	26,6	26,1	9,6	37,4	12,8	22,6	23,7
2	118,9	17,6	118,5	122,9	58,8	349,1	217,7	141,8	143,2
3	228,1	15,6	309,9	171,4	182,5	586,4	383,2	209,1	260,8
4	494,6	81,2	435,8	340,9	275,8	1079,7	522,9	385,3	452,0
5	470,0	123,3	503,5	705,9	1285,0	554,8	761,8	519,9	615,5
6	823,3	113,0	728,9	704,0	1296,8	887,2	880,8	934,2	796,0
7	1714,6	119,6	1009,6	1308,0	1192,7	918,8	920,8	1134,7	1039,8
8	1340,0	191,7	1618,0	880,8	1700,4	1377,4	1267,9	1375,5	1219,0

Table S3. Mice volume tumor size evolution (in mm^3) during 8 weeks of treatment for the DOTAU-Cl alone group.

**Phenazine #14**									
**Week**	**Mouse Tumor Volume in mm3**								**Mean**
	**1**	**2**	**3**	**4**	**5**	**6**	**7**	**8**	
1	36,6	17,9	26,6	26,1	9,6	37,4	12,8	22,6	23,7
2	118,9	17,6	118,5	122,9	58,8	349,1	217,7	141,8	143,2
3	228,1	15,6	309,9	171,4	182,5	586,4	383,2	209,1	260,8
4	494,6	81,2	435,8	340,9	275,8	1079,7	522,9	385,3	452,0
5	470,0	123,3	503,5	705,9	1285,0	554,8	761,8	519,9	615,5
6	823,3	113,0	728,9	704,0	1296,8	887,2	880,8	934,2	796,0
7	1714,6	119,6	1009,6	1308,0	1192,7	918,8	920,8	1134,7	1039,8
8	1340,0	191,7	1618,0	880,8	1700,4	1377,4	1267,9	1375,5	1219,0
**DOTAU**									
**Week**	**Mouse Tumor Volume in mm3**								**Mean**
	**1**	**2**	**3**	**4**	**5**	**6**	**7**	**8**	
1	19,1	38,9	24,7	0,0	0,0	46,9	31,5	32,7	24,2
2	282,1	198,1	284,3	147,3	170,2	268,0	377,6	317,2	255,6
3	169,9	225,3	482,7	764,6	212,1	145,7	464,1	308,4	346,6
4	227,8	354,7	596,8	941,3	224,7	1139,0	400,9	555,0	555,0
5	1286,1	552,8	472,5	1134,2	631,2	887,9	645,2	567,1	772,1
6	1007,8	565,0	497,4	1178,8	935,1	2294,4	955,6	864,0	1037,2
7	1777,4	956,4	1502,8	1381,0	1418,2	2304,1	1407,1	1345,3	1511,5
8	892,8	1686,5	1147,3	1950,8	1567,4	2656,1	2143,1	1953,7	1749,7

Table S4. Mice volume tumor size evolution (in mm^3) during 8 weeks of treatment for the $NP_{DOTAU\text{-}phenazine\ \#14}$ group.

**$NP_{DOTAU\text{-}phenazine\ \#14}$**											
**Week**	**Mouse Tumor Volume in mm3**								Mean	SD	SEM
	**1**	**2**	**3**	**4**	**5**	**6**	**7**	8			
1	19,9	42,4	18,6	6,0	10,2	48,2	0,0	31,5	22,1	17,3	6,1
2	117,0	189,8	101,3	52,1	174,3	99,5	28,7	88,4	106,4	54,9	19,4
3	211,0	287,1	190,7	87,9	231,9	372,0	44,6	145,5	196,3	105,5	37,3
4	240,9	426,3	393,0	209,1	512,0	237,8	45,8	147,7	276,6	155,2	54,9
5	335,3	567,8	199,6	274,6	463,1	394,2	59,5	156,3	306,3	167,9	59,4
6	418,4	548,0	455,3	614,3	403,4	405,1	79,2	202,6	390,8	174,2	61,6
7	562,7	753,0	583,2	837,6	570,0	409,6	123,7	213,6	506,7	246,5	87,1
8	1559,0	1077,8	602,1	1064,5	601,5	534,7	135,9	224,9	725,1	478,0	169,0
